# The Added Value of ^68^Ga-FAPI PET/CT in Patients with Head and Neck Cancer of Unknown Primary with ^18^F-FDG–Negative Findings

**DOI:** 10.2967/jnumed.121.262790

**Published:** 2022-06

**Authors:** Bingxin Gu, Xiaoping Xu, Ji Zhang, Xiaomin Ou, Zuguang Xia, Qing Guan, Silong Hu, Zhongyi Yang, Shaoli Song

**Affiliations:** 1 Department of Nuclear Medicine, Fudan University Shanghai Cancer Center, Shanghai, China;; 2 Department of Oncology, Shanghai Medical College, Fudan University, Shanghai, China;; 3 Center for Biomedical Imaging, Fudan University, Shanghai, China;; 4 Shanghai Engineering Research Center of Molecular Imaging Probes, Shanghai, China;; 5 Department of Radiation Oncology, Fudan University Shanghai Cancer Center, Shanghai, China;; 6 Department of Medical Oncology, Fudan University Shanghai Cancer Center, Shanghai, China; and; 7 Department of Head and Neck Surgery, Fudan University Shanghai Cancer Center, Shanghai, China

**Keywords:** ^68^Ga-FAPI, head and neck, cancer of unknown primary, metastases

## Abstract

^18^F-FDG PET/CT plays an important role in locating the primary tumor for patients with head and neck cancer of unknown primary (HNCUP). Nevertheless, in some cases it can be challenging to locate the primary malignancy on ^18^F-FDG PET/CT scans. Because ^68^Ga-radiolabeled fibroblast activation protein inhibitor (FAPI) PET/CT has promising results in detecting different tumor entities, our study aimed to evaluate the performance of ^68^Ga-FAPI PET/CT for detecting the primary tumor in HNCUP patients with negative ^18^F-FDG findings. **Methods:** Eighteen patients (16 men and 2 women; median age, 55 y; age range, 24–72 y) with negative ^18^F-FDG findings were enrolled in this study. All patients underwent ^18^F-FDG and ^68^Ga-FAPI PET/CT within 1 wk. Biopsy and histopathologic examinations were performed in the sites with positive ^68^Ga-FAPI PET/CT findings. **Results:** ^68^Ga-FAPI PET/CT detected the primary tumor in 7 of 18 patients (38.89%). Among these 7 patients, primary tumor sites included the nasopharynx (*n* = 1), palatine tonsil (*n* = 2), submandibular gland (*n* = 2), and hypopharynx (*n* = 2). The primary tumors showed moderate to intensive uptake of ^68^Ga-FAPI (mean SUV_max_, 8.79; range, 2.60–16.50) and excellent tumor–to–contralateral normal-tissue ratio (mean SUV_max_ ratio, 4.50; range, 2.17–8.21). In lesion-based analysis, 65 lymph nodes and 17 bone metastatic lesions were identified. The mean SUV_max_ of lymph node metastases was 9.05 ± 5.29 for ^18^F-FDG and 9.08 ± 4.69 for ^68^Ga-FAPI (*P* = 0.975); the mean SUV_max_ of bone metastases was 8.11 ± 3.00 for ^18^F-FDG and 6.96 ± 5.87 for ^68^Ga-FAPI (*P* = 0.478). The mean tumor-to-background ratios of lymph node and bone metastases were 10.65 ± 6.59 versus 12.80 ± 8.11 (*P* = 0.100) and 9.08 ± 3.35 versus 9.14 ± 8.40 (*P* = 0.976), respectively. **Conclusion:** We present the first evidence, to our knowledge, of a diagnostic role of ^68^Ga-FAPI PET/CT in HNCUP. Our study demonstrated that ^68^Ga-FAPI PET/CT has the potential to improve the detection rate of primary tumor in HNCUP patients with negative ^18^F-FDG findings. Moreover, ^68^Ga-FAPI had a performance in assessing metastases similar to that of ^18^F-FDG.

## Visual Abstract

**Figure:**
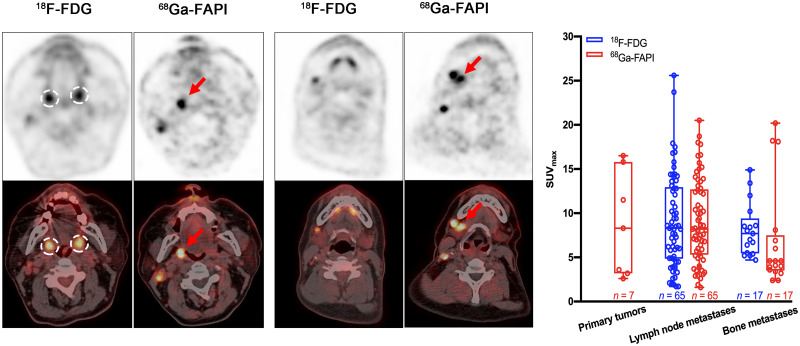

Head and neck cancer of unknown primary (HNCUP) is defined as a metastatic disease in the cervical lymph nodes with an unidentifiable primary tumor (*1*), even after a thorough diagnostic workup according to the National Comprehensive Cancer Network (*2*) and American Society of Clinical Oncology guidelines (*3*). HNCUP constitutes 1%–5% of all head and neck cancers (*4**,**5*). Squamous cell carcinoma (SCC) is the most common pathologic type of HNCUP, and approximately 90% of these cases are associated with human papillomavirus (*1*). The most frequent primary site of HNCUP is the oropharynx, accounting for 80%–90% (*6*). However, factors such as small tumor volume, hidden location, slow growth rate, and tumor involution hinder primary site identification (*7*). The absence of primary tumor identification may result in uncertain treatment decisions and increasing psychologic burden for patients with HNCUP (*8*).

Medical imaging plays an important role in oncology, particularly in tumor location (*9*). Conventional imaging modalities, CT and MRI, can provide plentiful anatomic information about primary and metastatic malignancies. However, the detection rates of the primary site for these 2 imaging modalities range from 9% to 23% in HNCUP (*10*–*12*). PET/CT, a typical molecular imaging modality, outperforms CT and MRI in identifying the primary tumor, with a detection rate of 25%–69% using ^18^F-FDG (*13*–*16*). Nevertheless, some limitations hamper the application of ^18^F-FDG PET/CT in primary tumor identification for HNCUP (*17**,**18*). First, physiologic ^18^F-FDG uptake can be seen in any lymphatic structure (especially Waldeyer’s ring), salivary glands, and brown fat. Second, uptake in the symmetric vocal cords and neck muscles is commonly seen if the patient talks or coughs during the uptake period. Third, infection and chronic inflammation (e.g., nasopharyngitis, amygdalitis, and gingivitis) can also result in high ^18^F-FDG uptake. These limitations may lead to false-positive findings, with a rate of 16%–25% (*4**,**13**,**16*). Last, false-negative ^18^F-FDG uptake can be seen in small, mucinous, well-differentiated, and necrotic lesions (*18*). Therefore, novel specific radiopharmaceuticals with low background uptake in the head and neck, which may better improve the detection rate of the primary tumor in HNCUP, are in urgent need.

Cancer-associated fibroblasts (CAFs), accounting for high proportion of most solid tumor mass, play a vital role in tumor growth, migration, and progression (*19*). The major feature discriminating CAFs from normal fibroblasts is the overexpression of fibroblast activation protein (FAP) (*20*). The presence of FAP was observed on a variety of epithelial and mesenchymal malignancies (*21**,**22*). Recently, ^68^Ga-radiolabeled fibroblast activation protein inhibitor (FAPI), a novel FAP-targeted PET tracer, has shown great value in the diagnosis of diverse carcinomas (*23**,**24*). Furthermore, studies (*25**,**26*) have demonstrated that ^68^Ga-FAPI revealed high uptake in primary tumors and low background noise in the head and neck region. These promising findings indicate that ^68^Ga-FAPI could serve as a potential alternative to ^18^F-FDG for the assessment of head and neck cancers.

Thus, the aim of this study was to investigate the value of ^68^Ga-FAPI PET/CT for identifying the primary tumor of ^18^F-FDG–negative HNCUP.

## MATERIALS AND METHODS

### Patient Selection

**FIGURE 1.**
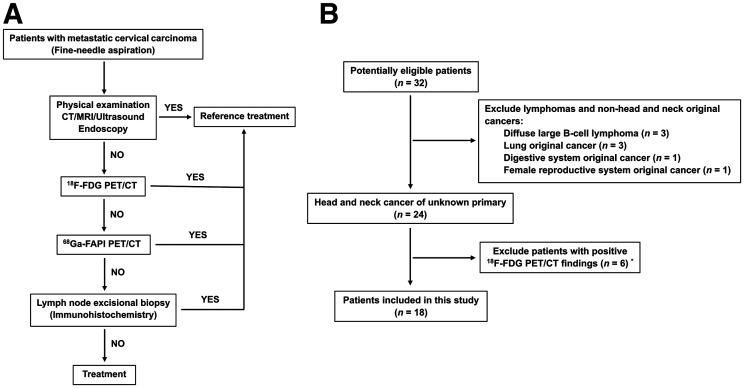
Flowchart of diagnostic workup (A) and patient selection (B). “YES” means the primary tumor was identified by these techniques and further confirmed by pathology, and “NO” indicates these techniques could not identify the primary tumor. *^68^Ga-FAPI PET/CT could also identify the primary tumor in these patients.

For patients whose primary tumor could not be identified by thorough medical history, clinical examination, medical imaging (e.g., contrast-enhanced CT, contrast-enhanced MRI, ultrasound, and ^18^F-FDG PET/CT), and endoscopy, ^68^Ga-FAPI PET/CT was recommended, based on the decision of a multidisciplinary team in head and neck cancer (Fig. 1A). In addition to patients in whom ^18^F-FDG findings were negative for localizing the primary tumor, ^68^Ga-FAPI PET/CT was also recommended to patients in whom ^18^F-FDG findings were positive for localizing the primary tumor who had undergone a biopsy that resulted in a negative finding. To further investigate the role of ^68^Ga-FAPI PET/CT in HNCUP, inclusion criteria were as follows: adult patients (age > 18 and < 80 y); pathology-confirmed metastatic cervical carcinoma by fine-needle aspiration; conventional imaging modalities (e.g., contrast-enhanced CT, contrast-enhanced MRI, or ultrasound) could not provide positive finding of primary tumor; both ^18^F-FDG and ^68^Ga-FAPI PET/CT were performed. The exclusion criteria were patients with lymphomas or non–head and neck original cancers, confirmed by immunohistochemistry; patients with both positive ^18^F-FDG and ^68^Ga-FAPI PET/CT findings for primary tumors, including anaplastic thyroid carcinoma, lymphoepithelioma-like carcinoma, and biopsy-negative but clinically diagnosed nasopharyngeal carcinoma; patients with 2 or more malignant tumors history; and patients unwilling to undergo ^18^F-FDG or ^68^Ga-FAPI PET/CT. ^18^F-FDG PET/CT reported negatively for localization of primary tumor in patients with HNCUP would be regarded as negative ^18^F-FDG PET/CT findings. This prospective study was approved by Fudan University Shanghai Cancer Center Institutional Review Board (ID 2004216-25) conducted in accordance with the 1964 Declaration of Helsinki and its later amendments or comparable ethical standards, and all subjects signed an informed consent form.

### Radiopharmaceuticals and PET/CT Scanning Procedure

^18^F-FDG was produced automatically using the Explora FDG_4_ module with cyclotron (Siemens CTI RDS Eclips ST) in our center. DOTA-FAPI-04 (Jiangsu Huayi Technology Co., Ltd.) was radiolabeled with ^68^Ga solution (elution from the ^68^Ge generator IGG100, Eckert & Ziegler) according to the procedure of Lindner et al. (*27*). The radiochemical purities of ^18^F-FDG and ^68^Ga-FAPI were both more than 95%.

^18^F-FDG PET/CT was performed first, and ^68^Ga-FAPI PET/CT imaging was then performed within 1 wk. For ^18^F-FDG PET/CT scanning, patients fasted at least 6 h, maintaining venous blood glucose levels under 10 mmol/L before ^18^F-FDG administration. This fasting process was not necessary for ^68^Ga-FAPI PET/CT scanning. After injection of with 260.64 ± 40.81 MBq of ^18^F-FDG or 143.71 ± 16.19 MBq of ^68^Ga-FAPI, patients were kept in a quiet environment for approximately 60 min before examination. All images were obtained on a Biograph mCT Flow scanner (Siemens Medical Solutions). PET image datasets were reconstructed iteratively using an ordered-subset expectation maximization iterative reconstruction by applying CT data for attenuation correction. Two experienced nuclear medicine physicians independently analyzed and interpreted the images masked, and they reached a consensus in the case of inconsistency.

Increased radioactivity of primary and metastatic lesions compared with the muscle background uptake was defined as being positive, verified by biopsy or follow-up. For quantitative analysis, maximum or mean of SUV (SUV_max_ or SUV_mean_) normalized to body weight was manually computed for primary and metastatic lesions and healthy tissues by drawing a 3-dimensional volume of interest. Meanwhile, the SUV_max_ ratio for primary tumor was defined as the quotient of the SUV_max_ of primary tumor and the contralateral normal tissue, and tumor-to-background ratio (TBR) for primary and metastatic lesions was calculated according to the formula TBR = tSUV_max_/bSUV_mean_, where tSUV_max_ is the SUV_max_ of the tumor lesion, and bSUV_mean_ is the SUV_mean_ of muscle. The size of primary and metastatic lesions was measured by CT.

### Statistical Analysis

All statistical analyses were performed using SPSS 25.0 (IBM). Means with SDs or medians with ranges were used to describe continuous characteristics. To compare the uptake of ^18^F-FDG and ^68^Ga-FAPI in metastatic lesions, 2-sample *t* tests were used. Two-tailed *P* values less than 0.05 were considered statistically significant.

## RESULTS

### Patients

A total of 32 patients were enrolled consecutively from our center from June 2020 to February 2021, and 18 patients were included for further analysis according to the inclusion and exclusion criteria (Fig. 1B). The basic clinical characteristics are presented in Table 1. Among the included 18 patients (16 men and 2 women; median age, 55 y; age range, 24–72 y), 2 (11.11%) were infected with Epstein-Barr virus, 6 (33.33%) were infected with human papillomavirus, 16 (88.89%) were pathologically diagnosed with cervical lymph node SCC, and 2 (11.11%) had adenocarcinoma.

**TABLE 1.** Patients Characteristics

Patient	Sex	Age (y)	EBV-DNA status	HPV status	p16 status	Pathologic type of cervical lymph node
1	M	52	P	U	U	SCC
2	M	63	N	P	P	SCC
3	M	58	N	P	P	SCC
4	M	50	N	U	U	AC
5	M	41	U	P	P	AC
6	M	55	N	U	U	SCC
7	M	54	N	N	N	SCC
8	M	72	N	U	U	SCC
9	M	61	N	P	N	SCC
10	M	47	N	N	P	SCC
11	M	62	N	N	N	SCC
12	F	55	P	N	N	SCC
13	M	63	N	U	U	SCC
14	F	67	N	U	U	SCC
15	M	40	N	P	P	SCC
16	M	24	N	N	N	SCC
17	M	55	N	U	U	SCC
18	M	51	U	P	P	SCC

EBV-DNA = Epstein-Barr virus DNA; HPV = human papillomavirus; P = positive; U = unknown; N = negative; AC = adenocarcinoma.

### Comparison of ^18^F-FDG and ^68^Ga-FAPI PET/CT in Metastatic Lesions

**FIGURE 2.**
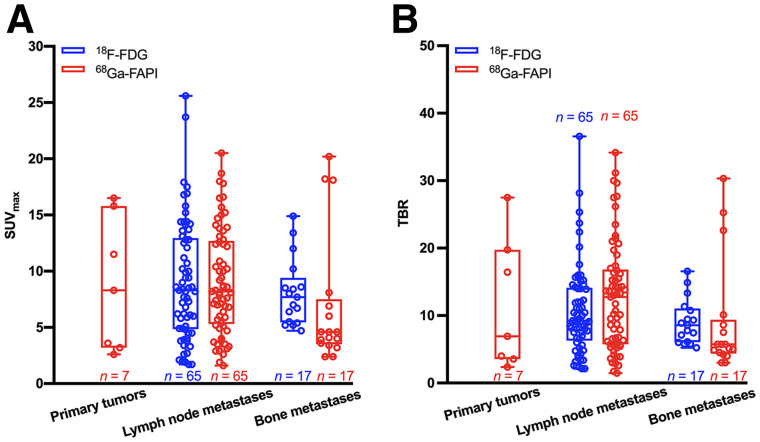
Box plots of SUV_max_ (A) and TBR (B) on ^18^F-FDG versus ^68^Ga-FAPI PET/CT.

A total of 65 lymph node and 17 bone metastases were detected by both ^18^F-FDG and ^68^Ga-FAPI PET/CT (Fig. 2, Table 2, and Supplemental Table 1 [supplemental materials are available at http://jnm.snmjournals.org]). Both tracers showed intensive uptake in lymph node and bone metastases. The mean SUV_max_ of lymph node metastases was 9.05 ± 5.29 for ^18^F-FDG and 9.08 ± 4.69 for ^68^Ga-FAPI (*P* = 0.975). The TBR for ^68^Ga-FAPI was a slightly higher than that for ^18^F-FDG (12.80 ± 8.11 vs. 10.65 ± 6.59, respectively); however, the difference was not significant (*P* = 0.100). For bone metastases, the mean SUV_max_ was 8.11 ± 3.00 for ^18^F-FDG and 6.96 ± 5.87 for ^68^Ga-FAPI (*P* = 0.478), and the mean TBR values were 9.08 ± 3.35 and 9.14 ± 8.40 (*P* = 0.976), respectively. Generally, no significant uptake difference was observed between ^18^F-FDG and ^68^Ga-FAPI in lymph node and bone metastases, indicating that ^68^Ga-FAPI PET/CT had a performance similar to that of ^18^F-FDG PET/CT in assessing metastases of head and neck cancers.

**TABLE 2.** Comparison of Metastatic Lesions on ^18^F-FDG and ^68^Ga-FAPI PET/CT in 18 Patients with HNCUP

	Metastases		Range of metastases size (mm)	^18^F-FDG		^68^Ga-FAPI		*P*	
Patient	Location	No.		SUV_max_	TBR	SUV_max_	TBR	SUV_max_	TBR
1	LN	3	7–8	5.27 ± 1.29	4.39 ± 1.07	2.27 ± 0.91	2.06 ± 0.82		
2	LN	2	7–20	6.55 ± 0.92	7.28 ± 1.02	7.10 ± 0.42	5.92 ± 0.35		
3	LN	2	10–16	8.10 ± 1.84	9.00 ± 2.04	14.40 ± 0.85	20.57 ± 1.21		
4	LN	16	7–22	10.78 ± 3.13	11.98 ± 3.48	13.69 ± 4.19	22.81 ± 6.98		
	Bone	1	N/A	8.00	8.89	18.20	30.33		
5	LN	8	4–8	2.70 ± 1.07	3.38 ± 1.33	9.41 ± 2.40	11.77 ± 3.00		
	Bone	1	N/A	8.60	10.75	20.20	25.25		
6	LN	2	17–22	5.60 ± 4.24	8.00 ± 6.06	5.35 ± 0.92	5.94 ± 1.02		
7	LN	1	17	7.60	8.44	12.80	14.22		
8	LN	1	38	25.60	36.57	15.20	19.00		
9	LN	4	7–17	15.78 ± 1.61	13.15 ± 1.34	3.83 ± 1.32	4.25 ± 1.47		
10	LN	1	27	7.30	9.13	3.10	2.58		
11	LN	2	13–20	15.35 ± 3.61	19.19 ± 4.51	5.60 ± 2.69	8.00 ± 3.84		
12	LN	3	13–21	10.80 ± 5.17	18.00 ± 8.62	5.63 ± 3.82	8.05 ± 5.46		
13	LN	1	10	6.20	8.86	8.60	9.56		
14	LN	8	5–11	6.81 ± 4.30	11.35 ± 7.17	7.60 ± 3.31	8.44 ± 3.68		
15	LN	1	5	2.10	2.63	2.90	3.63		
16	LN	3	12–26	12.63 ± 2.29	14.04 ± 2.55	11.87 ± 3.27	14.83 ± 4.08		
	Bone	15	N/A	8.08 ± 3.19	8.98 ± 3.55	5.33 ± 3.86	6.66 ± 4.83		
17	LN	3	10–19	8.80 ± 1.28	8.00 ± 1.16	7.60 ± 0.50	15.20 ± 1.00		
18	LN	4	4–18	11.08 ± 9.02	11.08 ± 9.02	7.58 ± 3.51	9.47 ± 4.39		
Sum	LN	65	4–26	9.05 ± 5.29	10.65 ± 6.59	9.08 ± 4.69	12.80 ± 8.11	0.975	0.100
	Bone	17	N/A	8.11 ± 3.00	9.08 ± 3.35	6.96 ± 5.87	9.14 ± 8.40	0.478	0.976

PET semiquantitative parameters were presented as means with SD.

TBR = tumor-to-background ratio; LN = lymph node; N/A = not applicable.

### ^68^Ga-FAPI PET/CT Imaging Results of Primary Tumors

**FIGURE 3.**
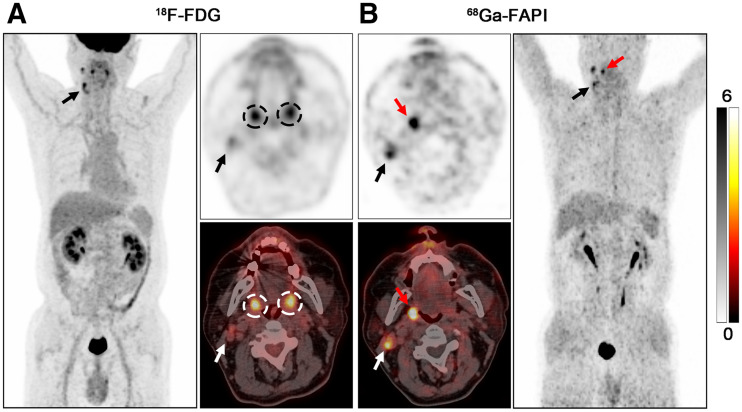
PET/CT scans with ^18^F-FDG (A) and ^68^Ga-FAPI (B) in 63-y-old male patient (patient 2) with metastatic SCC of right neck. ^18^F-FDG PET/CT was negative for detection of primary. Increased uptake of ^18^F-FDG was detected in palatine tonsils of both right and left sides (A, black and white dashed circles; SUV_max_ = 6.40 and 6.00, respectively), resulting an SUV_max_ ratio of 1.07. On ^68^Ga-FAPI PET/CT, there was asymmetric fullness with intensive uptake in right palatine tonsil (B, red arrow; SUV_max_ = 8.30), whereas low background uptake was seen in left palatine tonsil (SUV_max_ ratio = 3.46). Subsequent tonsillectomy confirmed SCC. Black and white arrows indicate metastatic lymph nodes.

**FIGURE 4.**
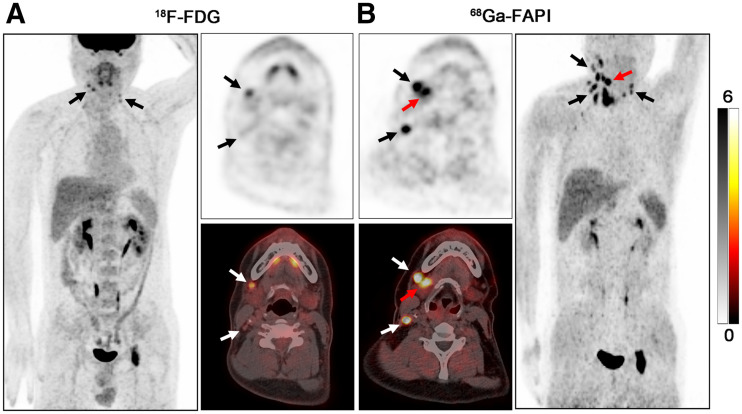
PET/CT scans with ^18^F-FDG (A) and ^68^Ga-FAPI (B) in a 41-y-old male patient (patient 5) with metastatic adenocarcinoma of right neck. ^18^F-FDG PET/CT was negative for detection of primary. On ^68^Ga-FAPI PET/CT, there was intensive uptake in right submandibular gland (B, red arrow; SUV_max_ = 15.80), whereas low background uptake was seen in left submandibular gland (SUV_max_ ratio = 6.87). Subsequent surgery confirmed salivary ductal carcinoma. Black and white arrows indicate metastatic lymph nodes.

Primary tumors in 7 of 18 (38.89%) patients with ^18^F-FDG–negative results were identified by ^68^Ga-FAPI PET/CT and pathologically confirmed by subsequent biopsy. ^68^Ga-FAPI PET/CT showed a higher detection rate in adenocarcinoma (2/2, 100%) than in SCC (5/16, 31.25%). Primary sites included the nasopharynx (*n* = 1), palatine tonsil (*n* = 2) (Fig. 3), submandibular gland (*n* = 2) (Fig. 4), and hypopharynx (*n* = 2) (Supplemental Fig. 1 and Table 3).

**TABLE 3.** Primary Tumor Characteristics and Semiquantitative Parameters of ^68^Ga-FAPI PET/CT

Patient	TNM	Primary tumor location	Pathologic type	Tumor size (mm)	^68^Ga-FAPI		
					SUV_max_	TBR	SUV_max_ ratio
1	T1N1M0 Stage II	Nasopharynx top wall	NDSCC	6 × 5	2.60	2.36	2.17
2	T1N1M0 Stage I	Palatine tonsil right side	SCC	11 × 10	8.30	6.92	3.46
3	T1N1M0 Stage I	Palatine tonsil right side	SCC	13 × 10	11.50	16.43	8.21
4	T2N2M1 Stage IVC	Submandibular gland right side	SDC	23 × 20	16.50	27.50	4.58
5	T1N2M1 Stage IVC	Submandibular gland right side	SDC	17 × 13	15.80	19.75	6.87
6	T1N1M0 Stage III	Hypopharynx posterior wall	SCC	5 × 3	3.20	3.56	2.91
7	T1N1M0 Stage III	Sinus piriformis right side	SCC	6 × 5	3.60	4.00	3.27

NDSCC = nonkeratinizing differentiated squamous cell carcinomas; SDC = salivary ductal carcinoma.

American Joint Committee on Cancer (AJCC) TNM stages for the 7 patients with ^18^F-FDG–negative results ranged from I to IVC (eighth edition of the AJCC TNM staging system) (*28*). The smallest primary tumor size detected by ^68^Ga-FAPI PET/CT was 5 × 3 mm. The mean SUV_max_ of ^68^Ga-FAPI for primary tumors was 8.79 (range, 2.60–16.50), and the mean TBR value was 11.50 (range, 2.36–27.50). When compared with the contralateral normal tissue, the primary tumor showed a remarkably higher uptake of ^68^Ga-FAPI, with a mean SUV_max_ ratio of 4.50 (range, 2.17–8.21).

## DISCUSSION

Identifying the primary tumor remains a concern for patients with HNCUP, though the development in imaging, endoscopy, and pathology techniques has progressed quickly. When no positive findings are obtained using noninvasive procedures, invasive diagnostic procedures such as tonsillectomy are then performed; these invasive procedures have a risk of bleeding or infection (*5*). Thus, noninvasive methods may be needed for improving the detection rate of primary tumor in HNCUP patients. This study investigated the performance of ^68^Ga-FAPI PET/CT in identifying the primary tumor of ^18^F-FDG–negative HNCUP. Our results demonstrated that ^68^Ga-FAPI can dramatically improve the detection rate of primary tumor in HNCUP patients compared with ^18^F-FDG. Furthermore, ^68^Ga-FAPI may show a performance similar to ^18^F-FDG in assessing metastases.

In the current study, the detection rate of the primary tumor by ^68^Ga-FAPI PET/CT was 38.89% (7/18). Notably, these patients all exhibited false-negative ^18^F-FDG PET/CT findings. Sites of false-negative ^18^F-FDG PET/CT findings included the nasopharynx, palatine tonsil, submandibular gland, and hypopharynx; these sites are different from previously reported observations that the tonsil was the most frequent false-negative location (*16*). Recently, Serfling et al. (*26*) reported ^68^Ga-FAPI PET/CT showed a better visual detection of the malignant primary in Waldeyer’s tonsillar ring than ^18^F-FDG PET/CT. However, the representative cases could provide positive findings of the primary site by ^18^F-FDG PET/CT alone in terms of HNCUP. Another study demonstrated that an SUV_max_ ratio of ^18^F-FDG uptake between tonsils of ≥1.6 could be regarded as malignancy and used to guide biopsy (*29*). In this study, 2 patients were diagnosed with palatine tonsil carcinoma by tonsillectomy. Puzzlingly, ^18^F-FDG PET/CT revealed no visual difference between right and left palatine tonsils in both cases. Furthermore, the SUV_max_ ratios of ^18^F-FDG uptake were all approximately equivalent to 1.00 (1.07 and 1.04 for patients 2 and 3, respectively), which was mistaken as physiologic uptake. By contrast, ^68^Ga-FAPI PET/CT showed intensive uptake in the tumor site and low uptake in the normal site, resulting in a visual difference (SUV_max_ ratio = 3.46 and 8.21, respectively). In line with our results, Syed et al. (*25*) demonstrated high ^68^Ga-FAPI avidity within tumorous lesions and low background uptake in healthy tissues of the head and neck region, again emphasizing the potential role of ^68^Ga-FAPI PET/CT in detecting palatine tonsil carcinoma, particularly in patients with ^18^F-FDG–negative results.

In addition to high-grade physiologic uptake in the head and neck, small lesion size was the major reason for the false-negative ^18^F-FDG findings due to the partial-volume effect and low tumor glucose metabolic activity (*30**,**31*). In the current study, ^18^F-FDG PET/CT missed 3 of 7 primary tumors because of their small size (diameter < 10 mm). Encouragingly, ^68^Ga-FAPI PET/CT revealed moderate uptake (SUV_max_ = 2.60, 3.20, and 3.60 for patients 1, 6, and 7, respectively) and a clearly visual difference (SUV_max_ ratio = 2.17, 2.91, and 3.27, respectively) in these primary tumors with small size, which was consistent with previous research (*24*). ^68^Ga-FAPI uptake is primarily based on the expression of FAP on CAFs in a solid tumor microenvironment, and even small T1 stage primary tumors could show a moderate FAP expression (*26*). Thus, to reduce the false-negative results by ^18^F-FDG PET/CT, ^68^Ga-FAPI could serve as an alternative tracer for identifying small primary tumors.

Most of the research focuses on SCC, as it is the most frequent pathologic type of HNCUP (*3*–*5*). However, other pathologic types, such as adenocarcinoma and neuroendocrine carcinoma, may cause diagnostic difficulties in clinical practice because of the lack of information regarding these pathologic types. Moreover, for cervical metastatic adenocarcinoma, diagnostic resection of the salivary gland is not recommended even after thorough noninvasive investigations. Furthermore, salivary gland cancers show a paucity of ^18^F-FDG avidity (*32*), which was proven again in our study (patients 4 and 5). Several non–^18^F-FDG radiopharmaceuticals, for example, ^18^F-fluorothymidine, ^68^Ga-DOTA-somatostatin analogs, and ^18^F-fluoromisonidazole, are recommended for detecting the primary tumor of HNCUP (*33*). However, these tracers are too specific to identify all types of head and neck cancers. Promisingly, recent studies have demonstrated ^68^Ga-FAPI can evaluate a broad spectrum of malignancies, including adenocarcinoma, neuroendocrine carcinoma, and well-differentiated carcinoma (*23**,**24*). In this study, ^68^Ga-FAPI showed intensive uptake in the submandibular gland (SUV_max_ = 16.50 and 15.80, respectively), providing sufficient information following surgery. Notably, ^68^Ga-FAPI had a higher detection rate in adenocarcinoma (2/2, 100%) than SCC (5/16, 31.25%) of HNCUP, indicating that ^68^Ga-FAPI was more sensitive to adenocarcinoma. However, further research with larger sample sizes is needed to verify this result.

Regarding the detection of regional and distant metastases, the performance of ^68^Ga-FAPI PET/CT varies among different studies (*24**,**26*). In our study, ^68^Ga-FAPI PET/CT showed a performance (*P* > 0.05) similar to that of ^18^F-FDG PET/CT in detecting both lymph node and bone metastases. Because radiation therapy is one of the most important modalities of treating HNCUP, the advantages of ^68^Ga-FAPI PET/CT in both primary tumors and metastases may play a vital role in gross tumor volume delineation.

There are some limitations in this study. The main limitation is the relatively small number of patients and that the number of pathologic types is imbalanced. In the future, larger population cohort studies with more cancer types need to be considered. Additionally, immunohistochemistry for FAP expression of primary tumors and metastases is lacking. Hence, FAPI imaging and FAP expression control studies are also necessary in the future.

## CONCLUSION

This study demonstrated that ^68^Ga-FAPI PET/CT can improve the detection rate of the primary tumor in HNCUP patients with negative ^18^F-FDG findings. Furthermore, for evaluating metastatic lesions, ^68^Ga-FAPI PET/CT showed a performance similar to that of ^18^F-FDG PET/CT. Because an improved detection rate is necessary in HNCUP, future research on more patients with HNCUP should be considered to evaluate the clinical value of ^68^Ga-FAPI PET/CT.

## DISCLOSURE

This work was funded by National Natural Science Foundation of China (grants 81771861, 81971648, and 81901778) and Shanghai Anticancer Association Program (grant no. HYXH2021004). No other potential conflict of interest relevant to this article was reported.

## Notes

KEY POINTS **QUESTION:** Does ^68^Ga-FAPI PET/CT have value for identifying the primary tumor in HNCUP patients with ^18^F-FDG–negative results? **PERTINET FINDINGS:** In this prospective study, ^68^Ga-FAPI PET/CT improved the detection rate (38.89%) of the primary tumor in HNCUP patients with negative ^18^F-FDG findings. **IMPLICATIONS FOR PATIENT CARE:** Our study provides a new strategy for identifying the primary tumor in patients with HNCUP, which may change their treatment decisions.
